# Characterization of recent thymic emigrants (RTEs), transitional B and Th17 cells in multiple sclerosis (MS)

**DOI:** 10.1186/1479-5876-9-S2-O2

**Published:** 2011-11-23

**Authors:** Aina Teniente-Serra, Laia Grau-López, María Jesús Martínez-Arconada, Marco Fernández-Sanmartín, Cristina Ramo-Tello, Ricardo Pujol-Borrell, Eva Martínez-Cáceres

**Affiliations:** 1 Immunology Laboratory, Banc de Sang I Teixits, Hospital Universitari Germans Triasi Pujol (HUGTP), Badalona, Spain; 2 Multiple Sclerosis Unit, Neurology Division, HUGTP, Badalona, Spain; 3 Citometry Unit, Institut d’Investigació en Ciències de la Salut Germans Trias i Pujol, Badalona, Spain; 4 Immunology Division, Hospital Universitari Vall d’Hebron, Barcelona, Spain; 5 Dept. of Cell Biology, Physiology and Immunology, Faculty of Medicine, Universitat Autònoma Barcelona, Spain

## 

The finding that APECED syndrome is due to a failure in negative thymic selection has suggested that central tolerance could play a role also in polygenic autoimmunity and that autoimmune patients should have an increase in RTEs. The aim of this work was to assess the frequency of RTEs, transitional B cells and Th17 lymphocytes in MS.

PBLs from 55 MS patients [20 relapsing-remitting (RR) with disease modifying drugs (DMD), 20 RRMS without DMD, 10 progressive MS and 5 RRMS patients on relapse] and 32 healthy controls were analysed by FACS for RTEs, naïve and memory T cells as well as Th17 cells and transitional B cells.

No differences in the percentage of RTEs (CD45RA+CD31+PTK7+) (3.30±2.94 vs 3.05±1.54, 0.933) or B transitional cells (CD19^+^CD24^hi^CD38^hi^CD27^-^) (8.67±3.62 vs 8.59±3.8, p=0.45) were found between controls and MS. A significant decrease in the percentage of CD4 effector memory (CD45RA-CCR7-CD27+) (23.13±8.76 vs 20.8±6.1, p=0.043), as well as an increase in Th17 cells (6.9±3.2 vs5.49±2.33, p=0.007) was observed in MS vs controls.

There were no differences in lymphocyte populations among the clinical forms of MS. RRMS under treatment had similar results to RRMS without DMD. Patients on relapse did show an increased proportion of CD4 effector memory cells, Th17 cells and RTEs and patients with higher EDSS had a higher percentage of Th17 cells (OR 2.3,p=0.045).

The results of this preliminary study do not favour a failure in central tolerance in MS but confirm involvement of Th17 cells which are associated with a higher degree of disability.

